# A Joint Venture: Advancing Health Equity for Underserved Communities Through Integrated Dermatology-Rheumatology Clinics

**DOI:** 10.7759/cureus.94590

**Published:** 2025-10-14

**Authors:** Zal Canteenwala, Joseph Thevathasan, Heli George Baho, Kunal Amin, Dimple Jain, Roshan Amarasena

**Affiliations:** 1 Dermatology, Shrewsbury and Telford Hospital National Health Service (NHS) Trust, Shrewsbury, GBR; 2 Medicine, Keele University, Shrewsbury, GBR; 3 Medicine, Epsom and St Helier University Hospitals National Health Service (NHS) Trust, Epsom, GBR; 4 Rheumatology, The Robert Jones and Agnes Hunt Orthopaedic Hospital, Oswestry, GBR

**Keywords:** dermatology, dermato-rheumatology, integrated care, nhs england, patient satisfaction, quality improvement, rheumatology, rural-based population, rural healthcare access, rural medicine

## Abstract

Background

Patients with immune-mediated disease often need both dermatology and rheumatology input. Separate appointments can increase travel and delay decisions, particularly in rural settings. We evaluated a monthly combined clinic in a rural UK catchment.

Methods

We conducted a prospective service evaluation (April-October 2022) of a consultant-led, co-located dermatology-rheumatology clinic. Forty-nine consecutive adult attendees completed an anonymous post-visit questionnaire on perceived usefulness, satisfaction, avoided appointments, travel costs, and prior time off work; free-text responses were thematically analysed by two reviewers. We report proportions with exact Clopper-Pearson 95% confidence intervals (CIs), with denominators varying due to item non-response.

Results

We analysed 49 questionnaires. All respondents viewed the joint appointment as a good idea (49/49; 100.0%; 95% CI 92.7-100.0), and all were satisfied (47/47; 100.0%; 95% CI 92.5-100.0). The clinic avoided an additional appointment for 44/46 (95.7%; 95% CI 85.2-99.5) and reduced out-of-pocket travel costs for 39/40 (97.5%; 95% CI 86.8-99.9). Among employed respondents, 19/36 (52.8%; 95% CI 35.5-69.6) reported previously needing time off work for separate specialty visits.

Conclusions

In a rural, cross-trust NHS setting, a combined dermatology-rheumatology clinic was feasible and associated with high patient-reported usefulness and satisfaction, fewer duplicate visits, and lower travel costs. Findings support continued provision and motivate comparative and economic evaluations using routine utilisation and cost data.

## Introduction

The prevalence of autoimmune conditions is rising in the UK. About 10% of people are affected, with rates varying by socioeconomic status and region [1]. These conditions often require input from several specialties. Different consultants see patients in separate clinics. That split increases travel, complicates scheduling, and delays decisions. These problems feel sharper given current NHS pressures.

Specialist services in rural England and Wales sit on multiple sites. In Shropshire and nearby areas, dermatology runs from the Royal Shrewsbury Hospital (RSH) with clinics at the Princess Royal Hospital, Telford (PRH). Rheumatology operates mainly from the Robert Jones and Agnes Hunt Orthopaedic Hospital (RJAH) in Oswestry, about 30 miles north-west of Shrewsbury. Patients who need both services often make repeated cross-county trips. Older adults and people who rely on limited public transport feel this most [2,3].

Care fragmentation (when treatment is split across clinicians and settings with limited continuity) has been linked to worse outcomes. In chronic illness, a recent systematic review found that greater fragmentation correlates with more emergency department attendances, additional (sometimes duplicated) tests, and higher healthcare costs [4].

For conditions that span specialties, co-located, coordinated clinics are a practical remedy: a single joint assessment, shared decisions, and an agreed plan reduce handovers, consolidate investigations, and streamline prescribing within one visit. Fragmentation also raises costs for both services and patients: providers absorb extra appointments and investigations, while patients shoulder longer journeys, time off work, and travel expenses (pressures felt most in rural, multisite services) [4]. Integrated clinics mitigate these pressures by using shared ordering and documentation (one clinic letter), which reduces duplication and accelerates decisions. With autoimmune disease and multimorbidity rising, collaboration is increasingly necessary.

In April 2022, we set up a joint dermatology and rheumatology clinic at RSH with colleagues from RJAH. Our a priori hypothesis was that a co-located, single-visit clinic would be associated with high patient-reported usefulness and satisfaction, consolidation of visits (i.e., avoidance of an additional appointment), and lower out-of-pocket travel costs; we also expected patients to report quicker, more coordinated decision-making. The clinic has a catchment covering Shropshire, Telford and Wrekin, and Powys in Mid Wales. Shropshire, home to both main hospitals, has 323,600 people across 1,346 square miles. Delivering care in that area is logistically demanding. The county is older than the UK average, with 24.7% aged over 65 compared with 18.6% nationally and 57% living in rural areas [2]. Its economy is mixed. Affluent neighbourhoods around Shrewsbury, a medieval county town of about 75,000, and some western districts sit next to pockets of deprivation in post-industrial and rural communities.

Powys lies to the west and has a very low population density, about 26 people per square kilometre, which places it among the most sparsely populated parts of Wales and the UK [5]. To the east is Telford and Wrekin, a unitary authority built around the planned New Town of Telford, designated in 1963 [6]. Its planned growth has created an urban centre within the wider West Midlands economy. Across the catchment, a clear rural-urban divide remains. Shrewsbury and Telford are the only major urban hubs among market towns and widely dispersed rural communities.

Dermatology and rheumatology meet often. Many conditions show both skin and musculoskeletal features [7]. Before the joint clinic, only 34% of patients with autoimmune disease saw both specialties, which exposed gaps in the traditional model [8]. Psoriatic arthritis shows the case clearly: treating skin plaques and joint inflammation together supports coordinated decisions about systemic therapy [8,9]. Most people with systemic lupus erythematosus have cutaneous disease, so collaboration improves management of skin and joint symptoms [10].

Other conditions benefit too. In dermatomyositis, assessing skin signs and muscle weakness at the same visit improves diagnostic accuracy. Vasculitis with skin involvement gains from the same approach [11,12]. The clinic also helps patients who develop cutaneous adverse effects from rheumatology drugs, particularly biologics and disease-modifying antirheumatic drugs (DMARDs).

Because of this geography and the need for integrated specialist care, we designed a prospective quality improvement evaluation of the combined clinic. We assessed patient-reported usefulness and satisfaction, and whether the joint visit avoided another appointment, reduced travel costs, and required time off work.

Findings from this service evaluation were accepted and presented as a poster at the British Association of Dermatologists (BAD) Annual Meeting, July 2025 (Glasgow, GBR).

## Materials and methods

Design and setting

We conducted a prospective service evaluation at RSH. The consultant-led joint dermatology and rheumatology clinic ran monthly from April to October 2022, giving seven clinics. The project was registered with the Trust audit department. Under local policy, this service evaluation did not require formal research ethics approval. Care followed usual pathways, and no experimental interventions were used.

Patients

Adults attending dermatology or rheumatology outpatient clinics were eligible if they had an autoimmune or inflammatory condition that required input from both specialties (for example, psoriasis with suspected or confirmed psoriatic arthritis, systemic lupus erythematosus with cutaneous disease, dermatomyositis, vasculitis with skin involvement, systemic sclerosis, or relevant overlap syndromes). We excluded those with predominantly non-autoimmune pathology and those unable to attend the joint clinic. Referrals arose from routine triage within existing outpatient pathways. No directly identifiable data were collected, and only limited non-identifiable demographics were recorded.

Clinic model

Each clinic provided a single same-day appointment with concurrent assessment by a consultant dermatologist and a consultant rheumatologist. Investigations and medicines were agreed in real time with shared ordering and medication review. Correspondence and results were issued jointly, and a unified plan was documented in a single clinic letter.

Data collection

Before leaving the clinic, patients were invited to complete a brief paper questionnaire with five yes/no items and an optional free-text box. The items asked whether (1) being seen jointly was a good idea (perceived usefulness), (2) they were satisfied with the appointment, (3) the combined format avoided an additional appointment, (4) the visit saved out-of-pocket travel costs, and (5) they had previously needed time off work for separate appointments. Completion was voluntary and anonymous; no direct identifiers were collected, in line with Trust information-governance policy.

We used a short, locally developed tool to minimise burden and fit the routine workflow in this preliminary evaluation. The goal was a rapid snapshot of satisfaction and access benefits, not comprehensive experience measurement with long validated scales. Collecting responses on-site reduced post-discharge non-response, supported patients without digital access, and enabled timely feedback.

During the evaluation period, medical students occasionally attended the joint clinics as part of routine placements. To capture informal educational impressions without adding burden to patients or clinic flow, we invited attending students to provide optional, anonymous free-text comments about perceived learning value (e.g., observations on seeing concurrent dermatology-rheumatology assessment and shared decision-making). No identifiable information was collected, no ratings were used, and these comments were not combined with patient data. Responses were summarised narratively to illustrate potential educational aspects of the model; they were not pre-specified outcomes and were not analysed using formal qualitative methodology.

Outcomes

Primary outcomes were the proportions answering yes to each of the five closed items. Free-text comments were analysed to contextualise and elaborate on the quantitative findings. The cost item asked whether the joint visit saved out-of-pocket travel costs; no other expense categories were collected.

Statistical analysis

Closed-item responses were summarised as counts and percentages. For each yes/no item, we report the proportion answering ‘yes’ with exact 95% confidence intervals (CIs) (Clopper-Pearson). Ambiguous markings (e.g., ‘?’ and crossed-out responses) and blanks were treated as missing; denominators, therefore, vary by item and reflect the number of non-missing answers. Free-text comments were analysed thematically using an inductive approach by two reviewers.

Reporting guideline

Reporting follows SQUIRE 2.0 (Standards for Quality Improvement Reporting Excellence). We describe the context, intervention, measures, study of the intervention, analysis, and ethical considerations in accordance with SQUIRE. A completed SQUIRE 2.0 checklist mapping items to manuscript sections is provided in the Appendices [13].

## Results

Quantitative findings

Forty-nine questionnaires were analysed. Outcome measures were overwhelmingly positive (Table 1), and overall satisfaction was 100% (47/47; 95% CI 92.5-100). All respondents viewed the joint appointment as a good idea (49/49; 95% CI 92.7-100). The combined clinic avoided an additional appointment for 44/46 (95.7%; 95% CI 85.2-99.5) and saved travel costs for 39/40 (97.5%; 95% CI 86.8-99.9).

**Table 1:** Primary outcome measures Values are n/N (%) with exact Clopper-Pearson 95% CIs. Denominators (N) vary due to item non-response; ambiguous markings and blanks were treated as missing. CI: confidence interval

Outcome	n/N (%)	95% CI
Joint clinic viewed as a good idea	49/49 (100.0)	92.7–100.0
Satisfied with the appointment	47/47 (100.0)	92.5–100.0
Avoided an additional appointment	44/46 (95.7)	85.2–99.5
Saved travel costs	39/40 (97.5)	86.8–99.9

Employment-related impact

The impact of clinic visits on employment was also analysed (Table 2). Among employed respondents, 19/36 (52.8%; 95% CI 35.5-69.6) reported previously needing time off work for separate visits.

**Table 2:** Employment-related impact Values are n/N (%) with exact Clopper-Pearson 95% CIs. Denominators (N) vary due to item non-response; ambiguous markings and blanks were treated as missing. CI: confidence interval

Measure	n/N (%)	95% CI
Previously required time off work for separate appointments	19/36 (52.8)	35.5–69.6
Did not require previous time off work	17/36 (47.2)	30.4–64.5

Qualitative findings

Several key themes emerged, which included comprehensive care, time efficiency, and cost savings (Table 3). Patients consistently praised the convenience and thoroughness of simultaneous specialist consultations. Free-text patient comments (students excluded) supported the survey findings and clustered into four themes (Table 3). The most frequent was coordinated, comprehensive care (12 mentions), with patients valuing ‘joined-up care’ and the ability to ‘talk to both consultants at the same time’. Time and decision efficiency (6) reflected perceptions of ‘get answers quicker’ and fewer repeat attendances (‘efficient’). Reassurance and professional confidence (5) highlighted the perceived thoroughness of joint assessment: ‘very reassuring’ and ‘very thorough’. Finally, travel and cost (4) pointed to fewer journeys across sites in this rural setting: ‘saves having to travel to Oswestry’. These themes align with the high satisfaction, fewer additional appointments, and reported travel savings in the quantitative data.

**Table 3:** Themes from patient free-text comments (patients only; students excluded)

Theme	Representative quotes (examples)	Mentions, n
Coordinated/comprehensive care	‘Gives joined-up care’; ‘able to talk to both consultants at the same time’; ‘same time for both skin and joints’	12
Time/decision efficiency	‘Get answers quicker’; ‘efficient’; ‘saves time for travel appointments’	6
Reassurance/professional confidence	‘Put mind to rest’; ‘very reassuring’; ‘very thorough’	5
Travel/cost benefits	‘Saves having to travel to Oswestry’; ‘travel to different hospitals’	4

Educational value (ancillary observation)

As an ancillary, descriptive component, brief free-text comments were obtained from three attending medical students (n = 3). They highlighted opportunities to observe cross-specialty assessment and joint decision-making in real time. Given the opportunistic, small, and non-validated nature of this feedback, and because it was not a pre-specified outcome, we present it solely as contextual information rather than an evaluative result.

## Discussion

This quality improvement initiative demonstrates the significant benefits of combined dermatology-rheumatology clinics. The unanimous positive feedback suggests that this model of care delivery effectively meets patient needs while potentially improving healthcare efficiency.

Geographical impact

Providing equitable care in rural Britain remains a challenge for the NHS and widens health inequalities. This problem is acute in areas with older populations, geographic isolation, and limited digital and transport infrastructure [14,15]. In 2019, the Royal College of Nursing warned that rural regions risk becoming ‘healthcare deserts’, affecting an estimated 10 million people [16].

Shropshire illustrates these pressures. Public transport has contracted sharply: regional data show a 63% fall in bus miles between 2015 and 2023, compared with a 19% national decrease [3]. Annual bus journeys fell from 5.7 million in 2012/13 to 2.4 million by March 2022, a 58% reduction. This matters for patients who must attend services on multiple sites, such as the RSH and the RJAH in Oswestry.

In our combined dermatology-rheumatology clinic, among respondents who answered the travel item, 39 of 40 (97.5%, 95% CI 86.8-99.9) reported saving money by making fewer journeys. Free-text comments echoed this finding; one patient noted that the model ‘saves having to travel to Oswestry’. Given the distance between sites and limited public transport, one combined visit often replaces separate trips to Shrewsbury and Oswestry, reducing fares and time away from work or caring.

These observations are consistent with analyses suggesting that urban-centred planning and resource allocation can miss rural needs, leading to suboptimal provision [17]. Service integration and the reduced travel it enables may help close part of that gap.

Clinical benefits

For patients with overlapping cutaneous-musculoskeletal disease, concurrent assessment by dermatology and rheumatology allowed fuller examination and a single, documented plan. Real-time discussion of skin and joint findings in one visit plausibly improves diagnostic confidence and treatment selection. Prior work reports refined or changed diagnoses in about one in five cases after joint specialist review [8]; our evaluation did not measure diagnostic change, but patient comments about ‘quicker decisions’ and ‘joined-up care’ are directionally consistent.

Accurate triage matters. Not all joint pain in psoriasis reflects psoriatic arthritis: one study found osteoarthritis alone in 27% and non-arthritic causes in 13% of such presentations [9]. Concurrent assessment can, therefore, reduce mislabelling and unnecessary immunosuppression.

Comorbidity management also benefits from a shared clinic. In one series, monitoring for hypertension and dyslipidaemia was required in 40% and 37% of patients, respectively [18]. Documentation quality improves in combined clinics: in separate clinics, rashes were noted in 5% of rheumatology notes and joint examinations in 1% of dermatology notes, whereas both were recorded in 100% of combined encounters [8]. In our rural setting, the same-day model additionally reduces repeat travel and access barriers.

Paediatric services report similar gains: all 13 surveyed North American centres cited better coordination and timelier communication, with high family satisfaction where measured [19]. Although not directly comparable to adult services, these findings support the generalisability of the approach.

Multispecialty models can extend further. The DERREGA unit (dermatology-rheumatology-gastroenterology) reported refined diagnoses in undifferentiated arthritis (psoriatic arthritis identified in 71.1% after dermatology input) and recognition of paradoxical psoriasis in roughly one-third of patients with inflammatory bowel disease [20], illustrating benefits where immune-mediated diseases co-cluster.

Service implementation

As a new service, the clinic received strongly positive feedback on coordination and convenience. One patient wrote, ‘combining the two clinics is a brilliant idea and very convenient, as we were able to talk to both consultants at the same time’. Another commented, ‘combining both services has to be more beneficial to both the hospital and its patients’.

Published evaluations of combined clinics report improvements in medication management; in one analysis, joint specialist review reduced total medications in 48% of cases through coordinated planning [8]. Our early experience is consistent with that pattern, with high satisfaction, smoother workflows, and clear educational value. The travel finding noted above was a consistent theme. Although these results are preliminary and context-specific, they can inform local service planning in rural settings. Larger, multisite evaluations with longer follow-up are needed.

Operational efficiency

Patients in our service reported fewer attendances and lower travel burden (avoided additional appointment 95.7% (44/46); saved travel costs 97.5% (39/40)). Prior evaluations also suggest efficient use of clinic time, with 96% of patients reporting receipt of ‘just the right amount’ of information [21]. Locally, there were early, informal signals of shorter waits for complex, multispecialty cases, consistent with more efficient use of specialist time and streamlined administration; these hypotheses require confirmation with prospective operational metrics.

Interpretation and comparison with prior work

Our findings align with prior interdisciplinary clinic evaluations that focused on diagnostic refinement and coordinated therapeutics in dermatology-rheumatology settings [8,9]. Evidence from UK co-located and ‘one-stop’ models supports strong patient-reported usefulness and efficiency gains. In psoriatic disease, a Manchester combined dermatology-rheumatology clinic reported markedly higher satisfaction for the joint model (mean 4.91/5 vs. 2.85/5 in separate clinics), with 94% ‘very satisfied’ and 89% ‘very involved’ in decisions, highlighting acceptability to patients [21]. Beyond dermatology-rheumatology, a randomised trial in symptomatic breast care showed that one-stop pathways achieved the diagnostic plan in fewer visits than standard care, demonstrating visit consolidation in a UK setting [22]. National improvement case studies likewise describe reorganising assessment, investigations, and review into a single visit rather than three, with associated streamlining of administrative steps (e.g., letters per episode falling from 4.3 to 1.5) [23]. Although direct trials quantifying travel savings from co-located clinics are limited, UK analyses estimate average out-of-pocket travel/parking costs of approximately £5.52 per outpatient car journey, implying tangible patient savings when duplicate or sequential attendances are consolidated; national outpatient guidance similarly reports substantial reductions in patient travel time and costs when attendances are rationalised [24,25]. Collectively, these UK data indicate that co-located, coordinated clinics are highly valued by patients, reduce duplicate visits, and plausibly lower travel costs by limiting the number of journeys.

The present work adds two elements that are under-reported: (1) delivery across two NHS providers serving a largely rural catchment and (2) quantified patient-reported access benefits-visit consolidation (95.7% avoided an extra appointment) and out-of-pocket travel savings (97.5%). Taken together, this suggests the model is not only clinically acceptable but operationally advantageous where geography, multisite services, and limited transport amplify travel burden. The results, therefore, complement urban/tertiary experiences by demonstrating feasibility and patient-perceived efficiency in a rural, cross-trust context. Future work should test generalisability with comparative designs, link to routine utilisation/wait-time data, and include formal cost analyses.

Future applications

This model appears most valuable where decisions hinge on cross-system assessment and longitudinal monitoring (e.g., psoriasis/psoriatic arthritis and cutaneous lupus). Evidence and pathways support adding gastroenterology for inflammatory bowel disease (IBD)-psoriasis/psoriatic arthritis interfaces (DERREGA reported that 97% of gastroenterology referrals were IBD requiring dermatology input) [20]. Endocrinology may add value for autoimmune polyglandular syndromes, thyroid disease with cutaneous manifestations, and metabolic effects of systemic therapies, enabling shared monitoring for patients on biologics or immunosuppression. Any expansion should be accompanied by clear referral criteria, shared documentation, and agreed drug-monitoring protocols.

Study limitations

This single-centre evaluation ran over a short period, in a rural, cross-trust setting with a modest sample (n = 49), which limits precision and generalisability. Referral through routine triage may have selected for more complex or engaged patients. We measured outcomes immediately after the visit using voluntary on-site questionnaires, so response bias, social-desirability bias, and possible novelty/Hawthorne effects cannot be excluded. Item non-response produced varying denominators across outcomes; we, therefore, report per-item results.

We used a brief, non-validated questionnaire. That choice, which was pragmatic for early-stage service evaluation and to minimise clinic burden, could have introduced measurement error and ceiling effects and prevented direct comparison with standardised patient-reported experience measures (PREMs). Future work should incorporate validated PREMs and objective endpoints such as re-attendance rates, waiting times, duplicated investigations, and travel/time costs.

We did not include a comparator arm, a formal health-economic evaluation, or linkage to routine utilisation datasets, and we did not quantify diagnostic reclassification, treatment changes, or test reductions. The cost item captured only out-of-pocket travel costs (fuel, fares, and parking as interpreted by respondents); other costs (childcare, prescriptions, and lost earnings) and precise distance/time savings were not collected. The study was not powered for subgroup analyses (e.g., by rurality or deprivation), limiting equity-focused inferences.

Finally, educational feedback from medical students was opportunistic and ancillary. The very small convenience sample and lack of a validated instrument mean these comments should be viewed as being contextual rather than evidential.

## Conclusions

In this single-centre evaluation of a monthly joint dermatology-rheumatology clinic, all respondents reported high satisfaction. Most avoided an extra appointment and reported lower travel costs. Seeing both specialties together clarified diagnosis and informed treatment for complex immune-mediated disease (e.g., psoriatic arthritis and cutaneous lupus). The clinic runs across two NHS trusts serving a largely rural population, indicating feasibility. Limitations include the single site, modest sample, item non-response (varying denominators), and the absence of a comparator; therefore, causal and economic inferences cannot be drawn. Continued provision is reasonable alongside prospective evaluation using objective service metrics and a formal health economic analysis, and exploration of other multispecialty models (e.g., dermatology-gastroenterology and rheumatology-endocrinology).

## Appendices

**Table 4:** SQUIRE 2.0 checklist SQUIRE 2.0 checklist [13]. Content reproduced and lightly adapted from SQUIRE 2.0 with attribution; use permitted under the Creative Commons Attribution–NonCommercial 4.0 International license (CC BY-NC 4.0). SQUIRE: Standards for Quality Improvement Reporting Excellence

SQUIRE item	What to report	Where addressed in this manuscript
Title/Abstract	Indicate this is quality improvement/service evaluation; key elements of intervention, context, outcomes	Title; Abstract (Background/Methods/Results/Conclusion)
Problem description	Nature/importance of the local problem	Introduction (rural context; fragmented care)
Available knowledge	Summary of what is known	Introduction (prior combined-clinic literature)
Rationale	Why the intervention should work; theory/logic	Introduction (co-located decisions; reduced travel)
Specific aims	Purpose of this initiative	Introduction (penultimate paragraph)
Context	Setting, organisational factors, catchment	Design and setting; Geographical impact
Intervention	What was done, by whom, and when	Clinic model
Study of the intervention	Approach to assessing impact; internal validity	Design and setting (prospective service evaluation); Outcomes
Measures	Process/outcome measures; definitions; data quality	Data collection; Outcomes
Analysis	Quantitative and qualitative methods	Statistical analysis; Analysis (thematic)
Ethical considerations	Ethics/approvals; justification for QI designation	Ethics Statement and Conflict of Interest Disclosures
Results	Outcomes, process measures, contextual elements	Results (Quantitative findings; Employment; Qualitative)
Interpretation	Key findings and their meaning	Discussion (Clinical benefits; Operational efficiency)
Limitations	Limitations, potential for bias, generalisability	Study limitations
Conclusions	Usefulness and implications; next steps	Conclusions
Funding/other information	Funding, roles, conflicts	Ethics Statement and Conflict of Interest Disclosures
